# Detection and Tracking of Environmental Sensing System for Construction Machinery Autonomous Operation Application

**DOI:** 10.3390/s25134214

**Published:** 2025-07-06

**Authors:** Junyi Chen, Qipeng Cai, Xinhai Hu, Qihuai Chen, Tianliang Lin, Haoling Ren

**Affiliations:** 1 College of Mechanical Engineering and Automation, Huaqiao University, Xiamen 361021, China; chjy@hqu.edu.cn (J.C.); 23014080001@stu.hqu.edu.cn (Q.C.); 21014080091@stu.hqu.edu.cn (X.H.); chen.qihuai@163.com (Q.C.); happyrhlly@126.com (H.R.); 2 Fujian Key Laboratory of Green Intelligent Drive and Transmission for Mobile Machinery, Xiamen 361021, China

**Keywords:** construction machinery, autonomous driving, object detection, object tracking

## Abstract

There are a large number of unstructured scenes and special targets in the construction machinery application scene, which brings greater interference to the environment sensing system for Construction Machinery Autonomous Operation Application. The conventional mature sensing scheme in passenger cars is not fully applicable to construction machinery. By taking the environmental characteristics and operating conditions of construction machinery into consideration, a set of environmental sensing algorithms based on LiDAR for construction machinery scenarios is studied. Real-time target detection of the environment, trajectory tracking, and prediction for dynamic targets are achieved. Decision instructions are provided for upstream detection information for the subsequent behavioral decision-making, motion planning, and other modules. To test the effectiveness of the information exchange between the proposed algorithm and the overall machine interface, the early warning and emergency braking for autonomous operation is implemented. Experiments are carried out through an excavator test platform. The superiority of the optimized detection model is verified through real-time target detection tests at different speeds and under different states. Information exchange between the environmental sensing and the machine interface based on safety warning and braking is achieved.

## 1. Introduction

Construction machinery is widely used in mining, municipalities, mountains and forests, and some other earthwork construction operations. The location of construction machinery operation is relatively fixed, and the process often requires repeating the same action extensively, which may cause operator fatigue. Additionally, the severe operational impacts pose risks to operator health. Currently, the intelligentization of construction machinery has become an important trend of development [1]. As an important part of the intelligentization of construction machinery, the environmental sensing system will directly lead to the decision-making of the whole machine and further affect the safety of operation. At present, considerable research has been carried out on the sensing systems for automatic vehicles. However, construction sites present unique challenges: a large number of unstructured scenes and special targets in construction machinery application scenarios [2], as well as the stringent regulatory compliance requirements governing safety systems and sensing technology for construction machinery scenes needs further in-depth research.

LiDAR belongs to active vision sensors, with the advantages of far detection and high accuracy not affected by light conditions, and can work in all weather, as well as provide complete scene depth information of the surrounding targets, which has a wide range of applications in high-precision map localization, obstacle detection, target identification, and tracking. It is very suitable for the special application environment requirements of construction machinery. Currently, a large amount of research has been conducted on the environmental sensing based on LiDAR. Zhou and Tuzel proposed the pioneering work VoxelNet [3] for voxel processing based target detection methods, which divided the disordered 3D point cloud into regular voxel features to achieve classification detection and position regression of objects. Yan et al. proposed the SECOND [4] network based on the VoxelNet network, which solved the problem of invalid computation in the blank region and improved the computation speed to 26 fps. The PointPillars [5] network, proposed by Lang et al., for the first time adopted a special voxel division (Pillar), as a way to realize the conversion of three-dimensional features into two-dimensional features, which greatly accelerated the detection speed, with an average detection speed of 62 Hz, and the fastest speed can reach 105 Hz. The CenterPoint [6] network, proposed by Yin et al., introduces the concept of CenterNet [7], from 2D to 3D, for the first time, which enabled 3D target detection through center point detection.

While advanced sensing technologies, such as LiDAR, enable sophisticated environmental perception, their direct deployment within safety-critical control functions faces significant regulatory barriers. This study presents a semantic segmentation-based obstacle prediction framework as a research prototype for collision avoidance. This prototype focuses primarily on perceptual layer algorithms and operates outside the scope of industrial safety standards, such as ISO 13849 [8]Performance Level d or IEC 62061 SIL 2 [9], which define the stringent requirements for safety-related electronic control systems under regulations like the EU Machinery Directive (2006/42/CE) [10]. Certified safety components (e.g., laser scanners) necessitate comprehensive capabilities, including real-time fault diagnosis, guaranteed response times, and high levels of EMC/IP robustness—requirements that are inherently challenging for research-grade systems utilizing frameworks like ROS to guarantee fully fail-safe operation and to mitigate potentially hazardous false positives or negatives. Therefore, this work concentrates specifically on advancing perceptual algorithms for collision detection, acknowledging the distinct objectives and constraints between research prototypes and certified industrial safety systems. In point cloud target tracking, it is mainly categorized into point-based tracking, twin frame-based tracking, and detection-based tracking paradigms, the mainstream method in the field of autonomous driving is still the detection-based tracking paradigm. Luo et al. proposed the FaFNet [11] network, which combined the three modular tasks of detection, tracking, and prediction, and was able to run in real time and with good results. Weng et al. proposed the AB3DMOT [12] algorithm, which was a further extension of the 2D multi-target tracking algorithm to the 3D domain, achieving high computational efficiency with the simplest combination. The CenterPoint [6] network proposed by Yin et al. coupled detection with tracking, which in turn simplified the tracking task.

While advanced perception systems (e.g., LiDAR-based detection/tracking) provide environmental awareness, translating this awareness into safe operational decisions remains a critical challenge—particularly in construction machinery’s unstructured environments. Here, reinforcement learning (RL) emerges as a pivotal decision-making paradigm, yet traditional RL suffers from safety risks and sample inefficiency in safety-critical applications [13]. The FHCPL [14] security reinforcement learning framework was proposed to maximize the rewards while guaranteeing the absolute security of policies, providing reliable solutions for industrial high-risk scenarios. TAG [15] provides lightweight solutions for RL challenges such as sparse reward and high-dimensional continuous control through Gaussian process teacher model with confidence bootstrapping mechanism, and promotes the application of RL in data-scarce scenarios, such as robotics and autonomous driving. FISOR [16] utilizes the reachability analysis of security control theory to transform the hard security constraint (zero violation) in offline reinforcement learning into a feasible region identification problem, and achieves decoupled, efficient training through a novel energy-guided diffusion model. However, the process of identifying feasible regions itself may implicitly rely on or make assumptions about the distribution of rewards and behaviors, and the safety of AI has been controversial, with most of the issues of safety policy, safety complexity, and safety benchmarking [14] remaining to be resolved. Therefore, the starting point of this paper is a study of perceptual content and its combination, and safety-related content will not be introduced in the subsequent content.

Different from passenger cars, there are a large number of unstructured scenes and special targets in the construction machinery context, which brings greater interference to the environment sensing system; the original mature sensing scheme in passenger cars is not fully applicable to construction machinery. In this paper, the detection and tracking of dynamic targets of the environmental sensing of construction machinery scenarios is studied to obtain important environmental information for subsequent dynamic obstacle avoidance, multi-machine cooperation, automatic machine following, and automatic operation, among other tasks. In order to verify the output of the environmental sensing system, dynamic target trajectory prediction and safety warnings are implemented on the real construction machinery only as a perception work contribution, not a safety-certified solution; at the same time, a general attempt is made in automated construction machinery obstacle avoidance to verify the direction of the subsequent research.

## 2. LiDAR-Based Sensing System for Construction Machinery

### 2.1. Preliminary Consideration

**Figure 1:**
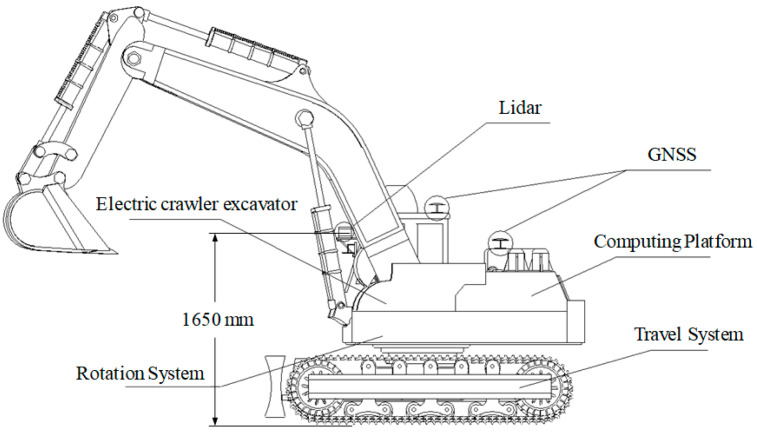
Schematic layout of the sensing system hardware.

The environment sensing system for construction machinery used in this paper is composed of LiDAR and the edge computing platform, as shown in Figure 1.

### 2.2. ROS-Based Sensing and Vehicle Control

**Figure 2:**
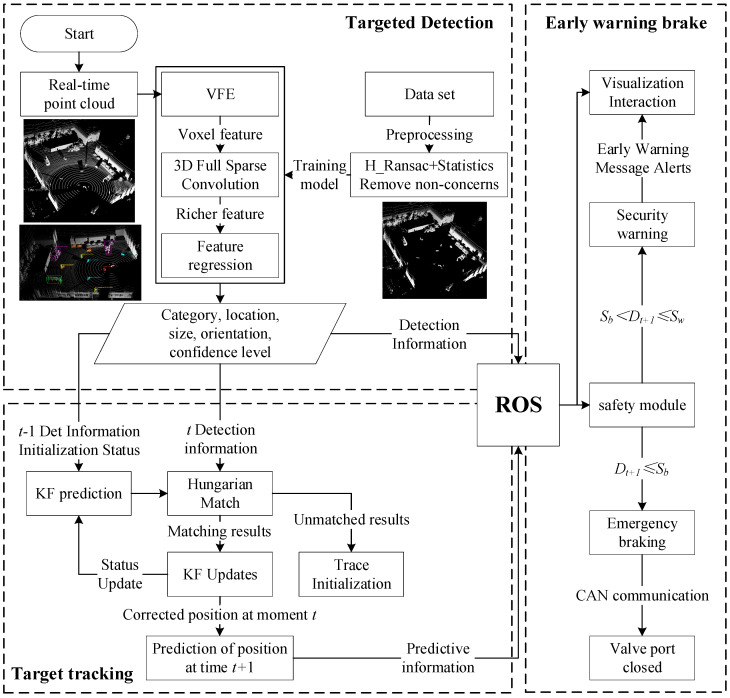
Sensing system software design program.

In order to realize the real-time communication between the environment sensing module and the planning and control modules, a distributed architecture Robot Operating System (ROS) is used [17]. The sensing system and the control strategy designed in this paper is given in Figure 2, which includes the target detection module, the target tracking module, and the whole machine interaction interface (namely the early warning braking module in this paper). The communication of multiple modules is realized through the ROS. When performing the sensing task, the detection module inputs the point cloud information scanned by LiDAR in real time, and outputs the information of the surrounding targets of interest, such as category, location, size, orientation, and confidence level, which is sent to the visual interactive interface for viewing through the ROS. When each frame detection task is carried out, the output detection information is input to the tracking module in real time. The tracking module uses the detection information of the previous frame to initialize the tracker, and predicts the prediction information of the current frame through the Kalman filter. Then, the Hungarian matching algorithm is used to match the detection information of the current frame with the prediction information. The corrected tracking information is obtained to realize continuous trajectory tracking of multiple targets in consecutive point cloud frames. Finally, the predictive ability of the tracker is used to obtain the target position of the next frame, and the prediction information is transmitted to the safety module through the ROS. The safety module starts to judge the safety distance after receiving the prediction information of each target of the next frame, and different warning safety thresholds and braking safety thresholds have been pre-set according to the different categories in the safety module. When the safety warning conditions are met, the module will send a warning prompt message to the visual interactive interface. When the emergency braking conditions are met, the module will send a reset message to the whole machine electric proportional pressure reducing valve through CAN bus communication. The corresponding emergency braking will be applied.

## 3. Detection Module

The environment sensing method based on deep learning is adopted in the detection module. Its performance is determined by large-scale high-quality datasets, excellent algorithm models, and high-performance hardware devices.

### 3.1. Dataset Construction and Processing

The detection effect of the model largely depends on a rich and high-quality dataset. Therefore, constructing a dataset specific to the construction machinery scenario is crucial for the study of target detection and target tracking tasks.

**Figure 3:**
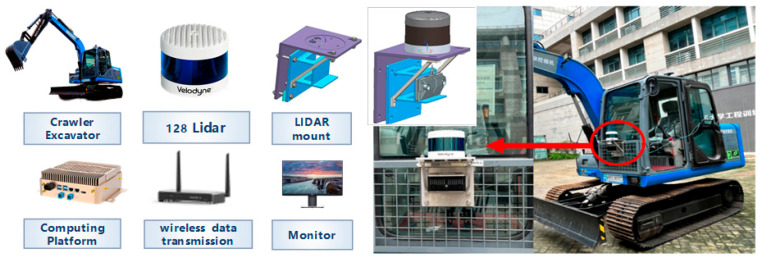
Data Acquisition Platform and Vehicle Arrangement.

At present, there is no mature open-source dataset in the field of construction machinery. The passenger car dataset cannot be applied to the construction machinery scenario directly. As a result, constructing a dataset specific to the construction machinery scenario is a prerequisite for the research of target detection and the tracking task. In this paper, we refer to the Kitti open-source dataset format and construct the required dataset based on the real-vehicle platform, as shown in Figure 3, which is the data acquisition platform and the layout of the real vehicle.

Two batches of point cloud data required for the training of deep learning algorithms are collected using the above data collection platform. In the first batch, point cloud data of seven scenes are collected, including point cloud data of different scenes of sunny daytime versus cloudy daytime and nighttime, as well as point cloud data of different walking modes, such as straight line round trip, driving in a circle, turning at right angles, and zigzag walking, among others. A total of 37,912 frames were obtained. Sensor failure problems caused by bad weather, such as rainy days, are not included in this study. The second batch of point cloud data is also collected for seven scenes, including common excavator behaviors such as traveling toward the truck, to the loading/unloading point, approaching pedestrians, and cockpit slewing. A total of 20,022 frames of point cloud data are collected. In order to be closer to the real operating scenes of construction machinery, and to facilitate repeated trials and tests, the data set chooses the courtyard where the construction machinery is parked as the simulation site. The environment in this data set is mainly simulated as a stockpile area and a narrow passageway.

**Figure 4:**
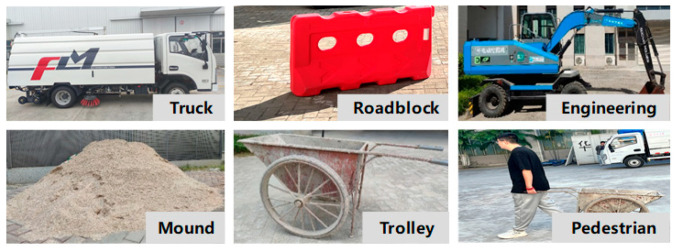
Data set labeling.

Considering that the sampling frequency of LiDAR used is 10 Hz, 5500 frames of point cloud data are selected from two batches of data for data labeling by interval frame sampling every 10 frames. The labeling categories are shown in Figure 4. The 5500 frames of point cloud data are divided into 4400 frames for the training set and 1100 frames for the validation set, according to the ratio of 4:1.

**Figure 5:**
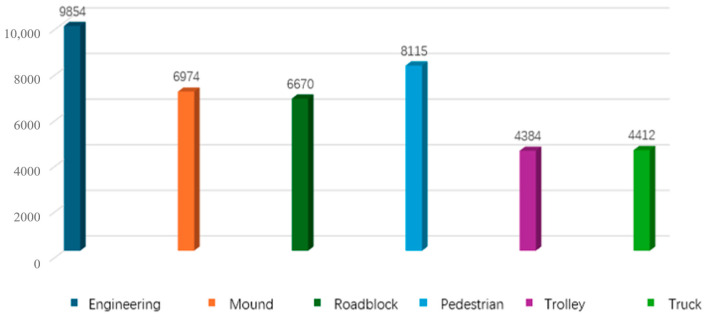
Distribution of data sets.

As shown in Figure 5, which reflects the distribution of the number of 3D point cloud frames in each category of this dataset, it can be seen from the figure that the number of samples in the dataset prepared in this study increases or decreases according to the complexity of the state of the object; thus, it has a certain balance and reasonableness, and it can satisfy the subsequent research of the task of target detection and target tracking.

The quality of the dataset greatly affects the training effect of the algorithm model. In the application of construction machinery, it is more about collaborating with other work equipment and/or staff. The demand for environmental sensing is relatively low for ground points in unstructured scenes. Therefore, in order to improve the quality of the data, to reduce noise and redundancy, to improve data distribution, and to optimize feature extraction, a random sampling method is employed with restricted a height threshold, which preprocesses the existing construction machinery scene dataset based on the random sample consensus (RANSAC) algorithm [18] combined with the restricted height threshold and statistical filtering.

**Figure 6:**
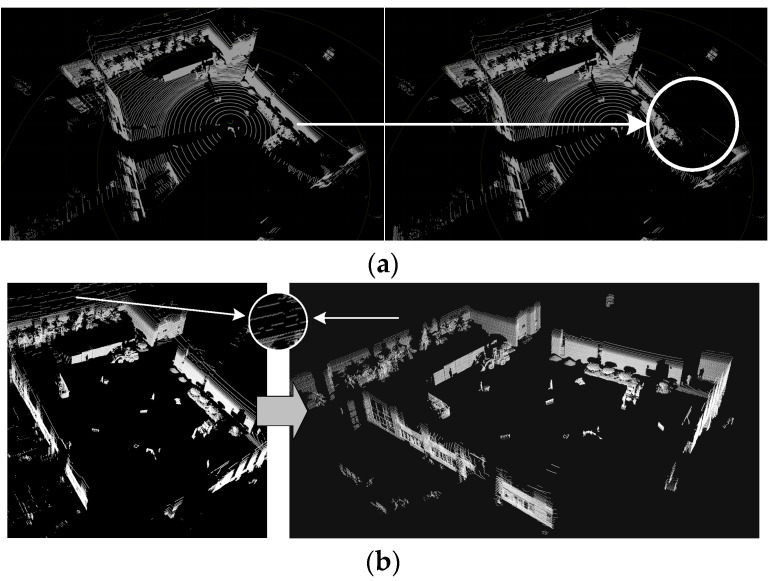
Point cloud preprocessing effect: (**a**) RANSAC error removes ground point cloud effect; (**b**) RANSAC combined with statistical filtering effects.

**Figure 7:**
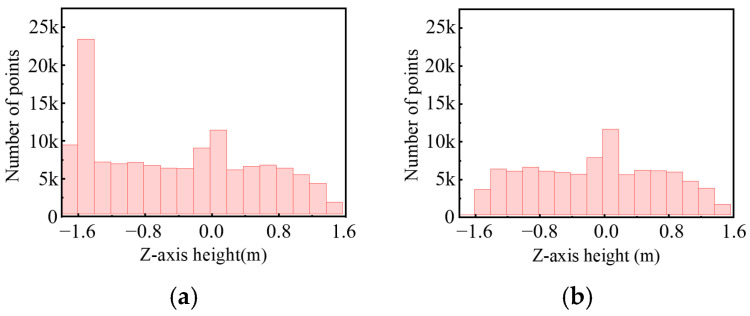
Statistics on the distribution of ground points before and after filtration: (**a**) pre-filter; (**b**) after filtration.

Since the RANSAC algorithm has randomness in initial point selection, it is prone to erroneous filtering when filtering ground points, as shown in Figure 6a. In order to select the correct initial points for plane fitting as much as possible, this paper filters the ground point cloud by restricting the height range of the selected points. To obtain the range interval where the ground points are located in the point cloud frames of the dataset, as shown in Figure 7, the height threshold of the selected points is obtained by counting the distribution of the points before and after the removal of the ground points of the point cloud in a single frame. The effect of the RANSAC algorithm combined with statistical filtering on the pre-processed point cloud data after limiting the height threshold is shown in Figure 6b. This approach not only accelerates the training speed of the algorithm model, but also facilitates the algorithm to better extract the feature information of the target points and improve the robustness and accuracy of the algorithm.

### 3.2. Detection Network and Accuracy Effects

**Figure 8:**
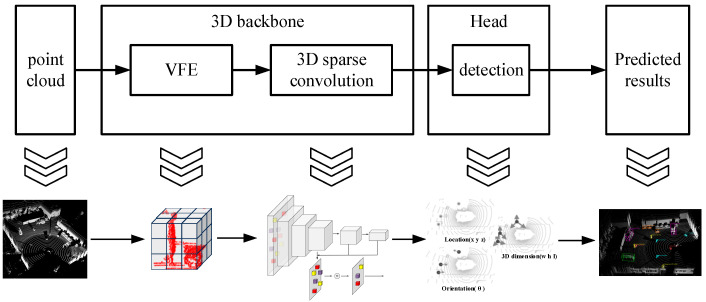
Point cloud 3D target detection network framework.

As shown in Figure 8, it is the point cloud 3D target detection network framework adopted in this paper, which includes three modules of voxel feature encoding (VFE), 3D sparse convolution, and multi-target detection header. After the original point cloud is input into the network, the unordered original point cloud is first divided into voxel squares of the same size according to a specific size [0.1, 0.1, 0.1] in the specified point cloud range [−43.2, −49.6, −2, 43.2, 49.6, 2] by the VFE module for feature encoding. The point cloud space is divided into 864 × 992 × 40 voxel grids that facilitates subsequent feature extraction of point cloud voxel features by the network.

The 3D sparse convolution module uses a combination of sub-fluid convolution and spatial 3D sparse convolution to form a convolution layer for feature extraction of the VFE feature N×C, which is converted to the standard voxel space of dimension N×5. Dimension 5 is [B,C,grid_z,grid_y,grid_x], respectively. The network sensory field is increased by means of multilayer convolutional layers, and the sparse features of the last two layers are combined into the fourth layer for summation at the same location by means of high compression, and transferred to the two-dimensional BEV sparse features.

Finally, the multi-target detection head module directly uses the 2D BEV sparse features output from 3D sparse convolutional compression to predict the confidence scores of the six types of voxels, the training is supervised by the Focal Loss function [19], the voxels closest to the center of the truth box are assigned as positive samples, and the sparse voxel features based on positive samples are supervised by the Loss function [20] to regress to obtain the bounding box size and location information. There is a multilayer perceptron (MLP) in each detection head, and the prediction information includes x,y,z,l,w,h,θ.

**Figure 9:**
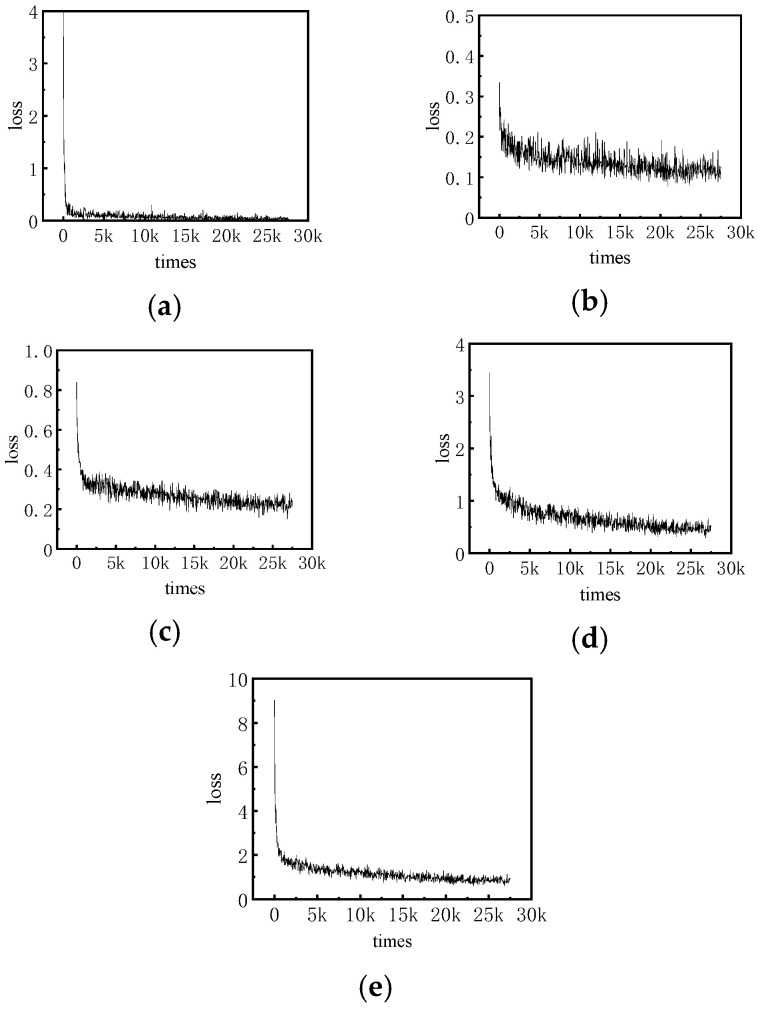
Loss change curve during training: (**a**) classification loss; (**b**) IOU loss; (**c**) DIOU loss; (**d**) localization loss; (**e**) total loss.

The 3D target detection network used in this paper is trained on 4400 frames of the self-made construction machinery training set during the training process. Due to the large scale of point cloud data, there is no need to image data like a large number of rounds of training before converging to the optimal. The number of training rounds is set to 50 epochs, and the batch size of the training is set to 8. It can be seen that a total of 27.5 K, as shown in Figure 9, is the change of the loss value during the training process.

Figure 9a–d shows the classification loss, IOU loss, DIOU loss, and localization loss of the target detection network, which correspond to the classification, the degree of matching between the prediction frame and the truth frame, the distance between the prediction frame and the truth frame, and the localization task, respectively; Figure 9e is the total loss curve, which is the weighted sum of the first four types of loss values. It can be found that the four types of loss function curves and the total loss curve at the beginning of training very rapidly decline, then began to enter the fine-tuning phase (tune-turning), and finally the trend is gradually smooth and finally stabilized at a smaller value near, indicating that the network has been converted to a more optimal state. The curve trend reflects the training process of the network as well as the number of training rounds of the setup of the rationality. The curve trend reflects the rationality of the training process and the setting of the number of training rounds.

In order to evaluate the effect of the trained model, AP and mAP, which are commonly used evaluation metrics for 3D target detection algorithms, are used to evaluate the model. The formulas are as follows [21]:

(1) $$AP{|}_{R_{40}}=\frac{1}{40}\sum_{r\in R_{40}}\underset{\tilde{r}\geq r}{\max}\rho (\tilde{r})$$

(2) $$mAP=\frac{1}{n}\sum_{i=1}^{N}AP_{i}$$

where $\rho (\tilde{r})$ is the precision at the recall equipartition point and $AP{|}_{R_{40}}$ is the mean value of the precision at the 40 recall equipartition points calculated.

Since the size and motion state of each category in the actual scene are different, in order to ensure the reasonableness of the evaluation, adhering to the principle of setting larger thresholds for easy-to-detect targets and smaller thresholds for difficult-to-detect targets, different evaluation thresholds are set for different categories. Table 1 shows the detection accuracy of this network training model in each category on the self-made construction machinery dataset.

**Figure 10:**
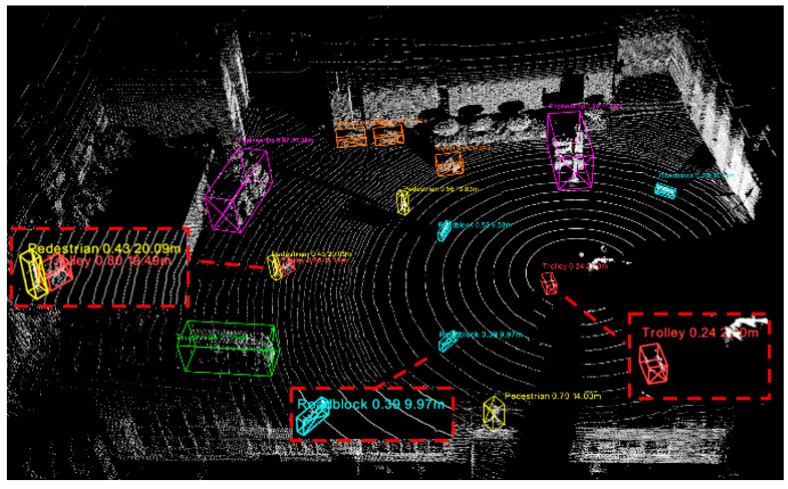
Visualization of target detection effects.

Figure 10 shows the detection effect of this 3D target detection network training model visualized in the demo, in which different color detection bounding boxes are set according to different categories and target categories, and confidence, distance, and other information are visualized. All targets appearing in the scene are all detected.

### 3.3. Detection Module Comparison and Ablation Test

(1) Comparison test

In order to verify the superiority of the performance of the 3D target detection network used in this paper, a comparison test between the network and the current mainstream four 3D target detection network detection effect are conducted, as shown in Table 2. The CenterPoint [6] algorithm is the feature encoding part of the two forms of Pillars and Voxel, therefore, the two participate in the algorithm at the same time to compare the detection accuracy. The methods involved in the comparison are PointPillars [5], SECOND [4], CenterPoint-V [6], CenterPoint-P [6], and VoxelNet [22], and the main comparison indexes are the mAP and the AP value of each category for each detection model trained for 50 rounds, and the network was trained by choosing adam as the optimizer, with the learning rate set to 0.003, weight decay to 0.01, momentum set to 0.9, and the learning rate tuning strategy to one cycle, setting each training batch size to 4.

In the models for comparison, the exact same dataset division was used and the same data-preprocessed point cloud data was used for training. As can be seen in Table 2, the 3D target detection algorithm used in the paper has an overall improvement in detection accuracy on the self-made construction machinery validation set compared to other algorithms. The detection algorithm in this paper improves 49.9% in detection accuracy for pedestrians, 4.4% for dump trucks, and 5.2% for mAP compared to the PointPillars algorithm. Compared to the VoxelNet [22] target detection network proposed by Chen et al. in 2023, our algorithm also improves 2.1% in overall detection accuracy for mAP. Compared to the CenterPoint [6] network based on CenterPoint detection, both in Voxel and Pillars form, it improves 24.4% and 12.5% in overall detection accuracy, respectively. From the comparison in the table, in the detection of the two categories of staff and dump truck, the detection accuracy of the network used in this paper is higher than that of other networks, which can be judged that the 3D target detection network used in this paper has better performance in the detection of the categories with more morphological changes and smaller size.

(2) Ablation test

In order to verify whether the operations used in this paper to optimize the performance of the 3D target detection network are effective and reasonable, ablation tests for different optimization operations on a self-made validation set of construction machinery are conducted.

There are three main measures to optimize the performance of the network, which are non-interest point removal for the dataset, increasing the IoU loss, and adjusting the weight coefficients. As shown in Table 3, the experiment is divided into four cases, the first case does not use any optimization measures and only relies on the original network trained on the homemade dataset without preprocessing, and the resulting detection model evaluates a mAP of 60.63 in the validation set. The second case employs the original network trained on the homemade dataset that has undergone the above mentioned dataset preprocessing operations, and the mAP is 75.38, which is an improvement of 24.3%. The third scenario modifies the loss function part for this 3D target detection network by adding the IoU loss function to help the model improve localization, with a mAP of 76.22, an improvement of 1.1%. Finally, the weight coefficients of the loss function are further adjusted, and the default weight coefficients of classification loss, regression loss, and intersection and parallel ratio loss of this network are 1.0, 2.0, and 2.0, respectively, and the best results are obtained by testing and adjusting them to be 1.0, 1.0, and 1.0, with the mAP of 76.93, which is an improvement of 0.93%. After the ablation test analysis, it shows that the three performance optimization operations used in this network have different degrees of enhancement to the network, and have a certain degree of effectiveness.

## 4. Tracking Modules and Early Warning Strategies

In practical operational scenarios, a single 3D target detection cannot meet the diverse sensing needs. Target detection is only limited to the environment sensing of single-frame data streams, but cannot support the connection between frames, so it is necessary to realize the sensing connection between multiple frames through 3D multi-target tracking.

### 4.1. Tracking Module

**Figure 11:**
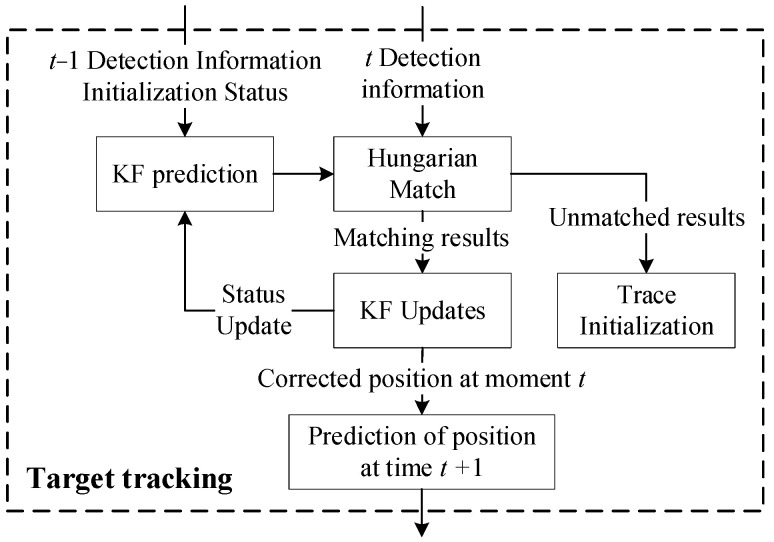
Target Tracking Algorithm Framework.

Detection-based tracking paradigm is still the dominant method in the field of autonomous driving, which is able to realize multi-target tracking well compared to point-based tracking and twin-frame-based tracking approaches without cumulative error, where detection and matching are being recalculated in every frame. The detection-based tracking paradigm is divided into three parts. including motion prediction, data association, and trajectory cycle management. Figure 11 shows the flow schematic of the tracking module in the sensing system of this paper.

(1) Movement Forecasting

Given that construction machinery (e.g., excavators ≤ 10 km/h, loaders ≤ 8 km/h) and surrounding targets (e.g., trucks ≤ 50 km/h, pedestrians ≤ 6 km/h) operate at consistently low speeds with minimal acceleration variations (typically |a| < 0.5 m/s^2^ during steady-state operations), we model their motion as a near-constant velocity. The Kalman filter algorithm is used for motion prediction.

The state prediction equation can be expressed as follows:

(3) $$\overset{\wedge }{x_{t}^{-}}=F\overset{\wedge }{x_{t-1}^{-}}$$

(4) $$P_{t}^{-}=FP_{t-1}F^{T}+Q$$

where $\overset{\wedge }{x_{t}^{-}}$ is the a priori estimate of the target state at moment t; $\overset{\wedge }{x_{t-1}^{-}}$ is the a posteriori estimate at moment t−1; F is the state transfer matrix; $P_{t}^{-}$ is the a priori covariance matrix at moment t; $P_{t}^{-}$ is the a posteriori covariance matrix at moment t−1; and Q is the process noise matrix.

The status update formula can be given as follows:

(5) $$K_{t}=P_{t}^{-}H^{T}{\left(HP_{t}^{-}H^{T}+R\right)}^{-1}$$

(6) $$\overset{\wedge }{x_{t}=}\overset{\wedge }{x_{t}^{-}}+K_{t}(Z_{t}-H\overset{\wedge }{x_{t}^{-}})$$

(7) $$P_{t}(I-K_{t}H)P_{t}^{-}$$

where $K_{t}$ is the Kalman gain matrix representing the weights; R is the observation noise matrix; $Z_{t}$ is the 3D detection information at moment t; H denotes the observation matrix of the system at a certain moment; I is the unit matrix; and $P_{t}$ is the a posteriori covariance.

State prediction is preceded by initialization of the state quantity x for the detection information output by the target point cloud input. As such, x can be expressed as follows:

(8) $$x=[x,y,z,l,w,h,\theta ,v_{x},v_{y}]$$

(2) Data Association

In order to make a connection between the previous and subsequent frames, it is necessary to correlate and match the historical frame prediction frame with the current frame detection frame. Firstly, the tracker is initialized with the previous frame detection information, and the current frame information is predicted by the Kalman filter. Then the similarity between the detected frame and the predicted frame is measured by the Mahalanobis distance, and the cost matrix is constructed. Then, the Hungarian algorithm is used to match the predicted frames with the detected frames in a cascade matching strategy, which prioritizes the detected frames to match with the predicted frames with a shorter vanishing time to avoid the frequent switching of tracking identifiers.

The Mahalanobis distance is not affected by the magnitude in the target detection information, and size, position, orientation, and other information magnitude are not the same. The Mahalanobis distance can be a good measure of the proximity between two points of information while ignoring the magnitude. The formula for the similarity measure of the Mahalanobis distance is shown as follows:

(9) $$d^{\left(1\right)}(i,j)={\left(d_{j}-y_{i}\right)}^{T}S_{i}^{-1}(d_{j}-y_{i})$$

where *d_j_* is the position of the *j*th detection frame, $y_{i}$ is the position of the ith prediction frame, and $S_{i}^{-1}$ is the covariance matrix between the detection and prediction frames.

(3) Trajectory cycle management

In order to avoid association matching error in multi-target tracking task, the prediction box is set to three states, including the tracking state, the pending tracking state, and the pending deletion state. When the detection frame is successfully matched with the prediction frame for the first time, it is in the pending tracking state, it starts the tracking counting, and turns to the tracking state if the number of matches reaches the threshold, and vice versa; when the prediction result fails to find a match for the detection frame, it enters into the pending deletion state, and deletes the target track if the number of losses reaches the threshold.

Different tracking thresholds and deletion thresholds are set accordingly for different dynamic and static target motion situations. The dynamic target motion situation is complex and variable, so larger thresholds are set, as shown in Table 4.

### 4.2. Security Alert Strategy

**Figure 12:**
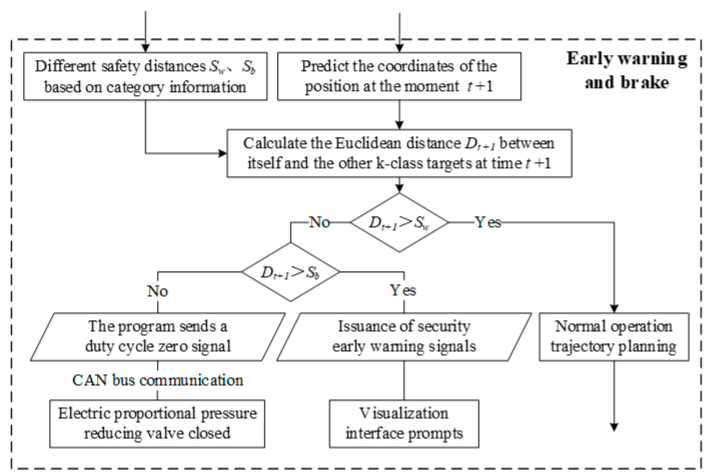
Early Warning Braking Strategy.

The early control strategy based on the safety distance model typically consists of two levels: early warning and braking. The first level involves issuing a warning when the relative distance between the self-vehicle and the target vehicle, measured by the information acquisition module, reaches the critical threshold. This triggers an audible warning to prompt the driver to brake. The second level involves braking, where the system tracks the target’s trajectory using sensory information, predicting its future position and relationship with the vehicle. By assessing whether the target will enter the warning or emergency braking zone, the system can issue an appropriate warning or braking signal. This ensures the safety of the electric crawler excavator during operations and multi-machine cooperation, preventing collisions and ensuring safe operation [23,24]. As a test of the perception module, we designed a simple warning strategy, as shown in Figure 12, which is a validation of our perception algorithm and confirms the feasibility of the perception algorithm.

In the selection of safety distances, Gounis and Bassiliades [25] simulated a three-layer AEB control system that evaluates collision risks by comparing the relative distance from the vehicle ahead with an adaptive speed-based distance threshold; and Park et al. [26] calculated the minimum distance for collision avoidance during braking and steering, taking into account the relative motion of surrounding vehicles and the lane information obtained through vision sensors. As shown in the above conventional methods, the safety distance can be dynamically adjusted in conventional vehicle driving scenarios after sensing the content, but the complexity of the construction machinery operating scenarios is far more than that of conventional vehicle driving scenarios; at the same time, its characteristics, such as low speed and fixed dynamic objects, allow for the use of a more fixed and safe safety distance. Therefore, the safety distance in this paper is determined by actual excavator experiments, and a relatively safe threshold is set.

Returning to the safety warning strategy, as shown in Figure 12, using the prediction information as an input, a warning braking strategy framework is constructed. First, according to the different categories of information to delineate the different warning safety distance $S_{w}$ and braking safety distance $S_{b}$, set the warning safety distance and braking safety distance to take into account that the excavator itself also exists in the safety radius S. At the same time, using the Euclidean distance to calculate the position of the next moment of its own position and the other k categories of targets. distance $D_{t+1}$, then determine whether the predicted distance $D_{t+1}$ is greater than the warning distance threshold $S_{w}$; if it is greater, then continue to maintain the use of real-time detection and tracking information for trajectory planning and other tasks, and vice versa. Then, enter into the next judgment logic, determine whether $D_{t+1}$ is greater than $S_{b}$; if it is greater, then send a warning signal to the visualization interface for prompting, and vice versa. Then, send an emergency stop signal and send a zero message to the proportional pressure-reducing valve through CAN bus communication to close the valve to achieve safety. The zero-setting message is sent to the electric proportional pressure reducing valve through CAN bus communication to close the valve port and realize safe emergency braking.

For the above warning and braking strategy, it is necessary to set different safety thresholds in advance according to different categories, as shown in Table 5, and the basis for setting the thresholds mainly refers to the statistics of the maximum length and width dimensions of each category of targets in the labeled dataset. Combined with the actual motion state of each type of target, the corresponding warning safety distance $S_{w}$ and braking safety distance $S_{b}$ are set for each category.

Construction machinery and trucks belong to large moving targets, taking into account the excavator’s own maximum digging radius, the two warning safety distance and braking safety distance are set to 9 m and 6 m, respectively The staff belong to small targets and the activity is larger, taking into account the redundancy of pedestrian safety, so the warning safety distance and the braking safety distance are set to 9 m and 4 m, respectively. The soil pile is an operational target, so only the warning safety distance is set to 6 m. The dump truck and the roadblock belong to medium-sized targets and are generally stationary, so the warning safety distance and the braking safety distance of the two are set to 6 m and 4 m, respectively.

### 4.3. Track Matching

**Figure 13:**
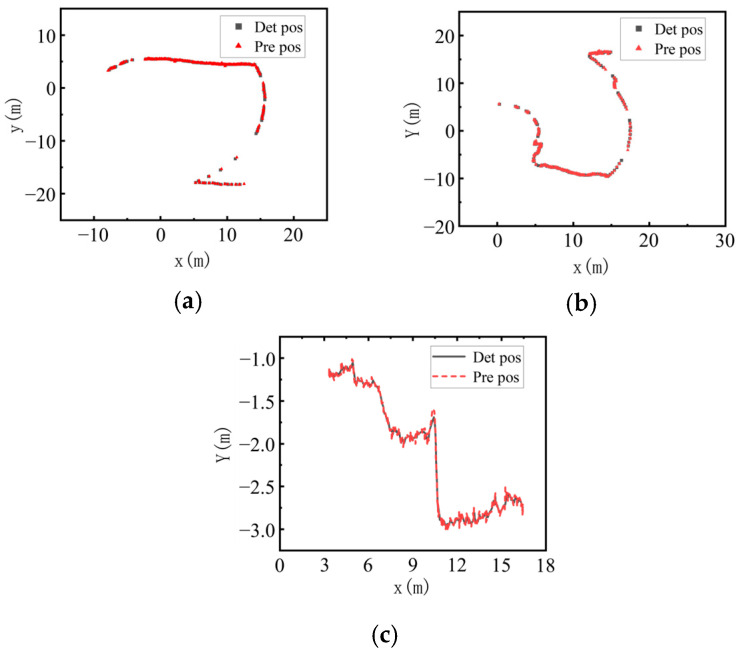
Global map of prediction and detection locations. (**a**) Engineering. (**b**) Pedestrian. (**c**) Truck.

In order to verify the effectiveness of the detection-based tracking algorithm for dynamic targets, three dynamic targets, construction machinery, workers, and trucks, were selected to observe the position fitting degree between their detected and predicted positions. In a 35 s continuous point cloud frame, since the actual scanning frame rate of LiDAR is 90% of the theoretical one, a total of 319 frames are obtained, and the number of frames that can be matched between the predicted and the detected positions is 318 frames. As shown in Figure 13, in the global map of the predicted and detected positions, the three types of targets show a good degree of fit, and the overall tracking effect is remarkable, which indicates that the algorithm used in this module has a certain degree of effectiveness.

## 5. Real-Vehicle Testing

**Figure 14:**
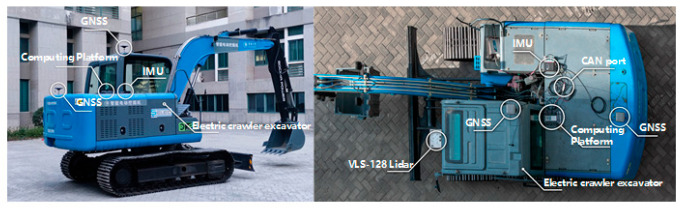
Vehicle test platforms.

In order to validate the effectiveness of the proposed sensing system, further real-vehicle tests are carried out for the accuracy, real-time performance, and dynamic safety warning of the detector, as shown in Figure 14 for the real-vehicle test platform. Table 6 shows the configuration parameters of the sensing system.

### 5.1. Verification of Detection Accuracy

There are a variety of working conditions and motion states in the actual driving operation scenarios; in order to verify the accuracy of the detector under different circumstances, the following two sets of tests are designed.

(1) Target detection test at different speeds

The electric crawler excavator used in this test has two motion modes, slow mode (turtle gear) and fast mode (rabbit gear). As shown in Table 7, the speed of the slow mode is 2.5 km/h, and the speed of the fast mode is 3.8 km/h, and the speed of the motion usually has a direct effect on the test.

**Figure 15:**
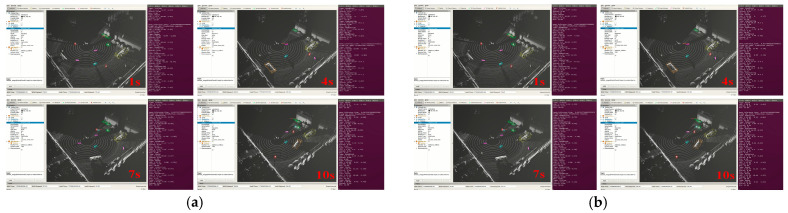
The effect of different speeds of real car testing: (**a**) the rapid mode real vehicle inspection effect; (**b**) the slow mode real car inspection effect.

As shown in Figure 15, traveling at two speeds for 10 s respectively, the point cloud frames at 1 s, 4 s, 7 s, and 10 s are selected to show the real vehicle detection results, and 20 frames of point cloud statistics of 10 s consecutive frames are extracted to show the real vehicle detection effect, as shown in Table 8.

The real-time target detection test conducted at different speeds shows that the average detection rate for different categories in the slow mode can reach 84.05%, and the same can have a high average detection rate of 82.03% in the fast mode.

(2) Target detection test under different motion states

Excavators in the actual working conditions usually have a variety of motion states, common motion states, including straight-line driving, turning, and cockpit rotation, among others, in the excavation operation will be rotated through the cockpit to adjust the digging position, as well as dumping soil loading and unloading, for these three types of common motion states for the real-time target detection test.

**Figure 16:**
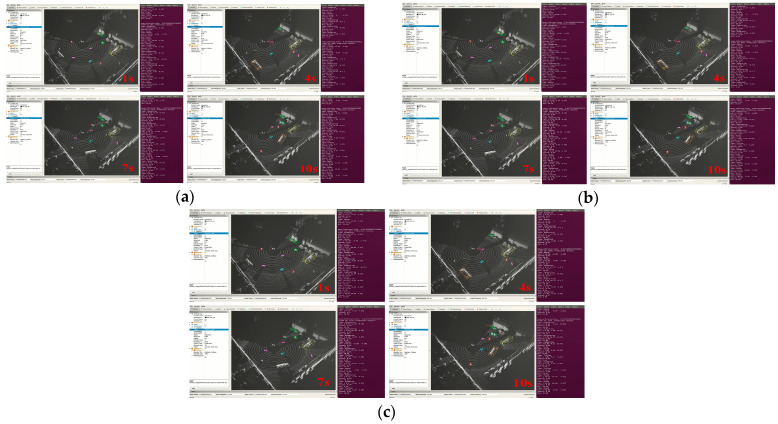
Real-time detection effect of different motion states: (**a**) real-time detection effect of straight-line driving.; (**b**) real-time detection effect of turn-by-turn driving; (**c**) cockpit rotation real-time detection effect.

As shown in Figure 16, the point cloud frames at 1 s, 4 s, 7 s, and 10 s are selected to show the real vehicle detection results after traveling for 10 s in the three motion states, respectively, and 20 frames of the point cloud in the consecutive frames of 10 s are extracted to statistically measure the detection effect of the real vehicle, as shown in Table 9.

The experimental results show that the target detection algorithm used in this study has more reliable detection performance in different speed modes and different motion states.

### 5.2. Detection of Real-Time Validation

The theoretical scanning frame rate of LIDAR in the sensing system is 10 Hz. To further verify the real-time situation from the sensor input to the point cloud input, validation experiments are carried out for two separate cases.

(1) Increase point cloud input frame rate

**Figure 17:**
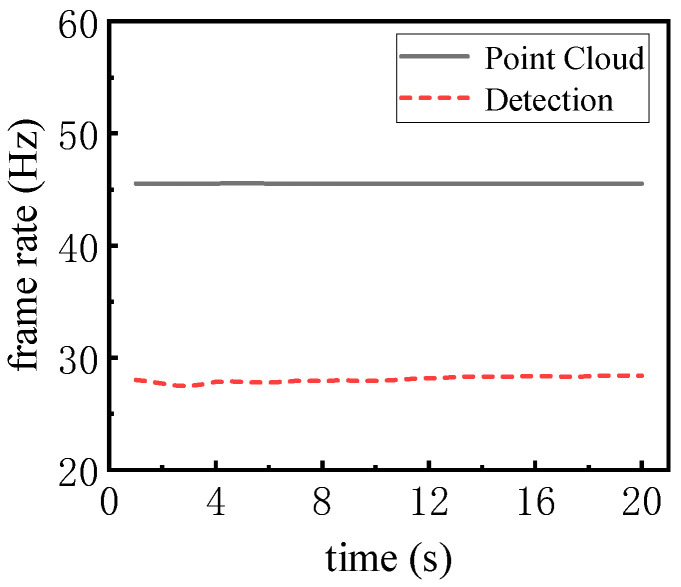
Comparison of point cloud input frame rate and detection frame rate.

In order to measure the limit detection frame rate of the detector, the frame rate of the point cloud input network is increased five-fold to measure the limit detection frame rate of the detector, as shown in Figure 17. The limit average detection frame rate of the detector in 20 s is 28.07 Hz, which is larger than the theoretical scanning frame rate of LiDAR.

(2) Real-time detection frame rate comparison

**Figure 18:**
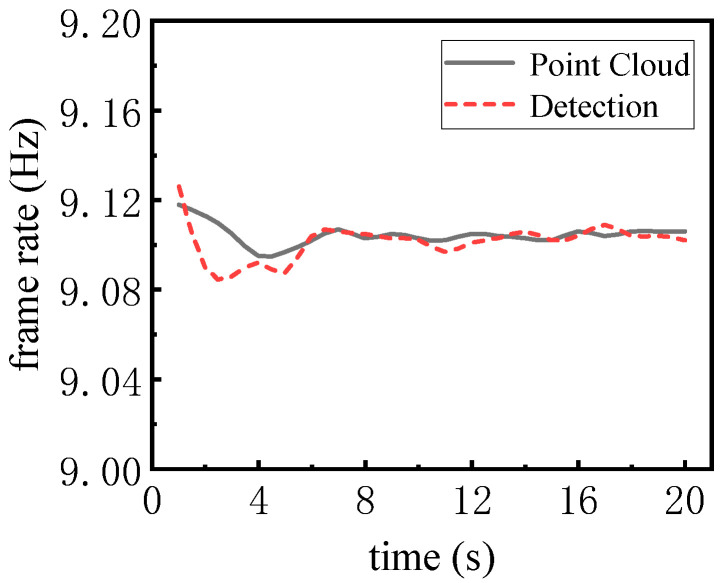
Comparison of real vehicle point cloud input frame rate and detection frame rate.

The theoretical scanning frame rate of LiDAR is 10 Hz, but the actual scanning to obtain the frame rate is only 90% of the theoretical value, so in order to verify the real-time situation of the actual operation of the detector, the real-vehicle point cloud input frame rate compared with the detection of the frame rate is shown in the Figure 18. It can be seen that as the detector is just started, there is a certain delay but, after 5 s, it tends to be stabilized.

The detection performance of the network can be judged by the two ways of the above test to meet the real-time requirements of the motorized crawler excavator during the actual walking operation.

### 5.3. Security Alert Policy Validation

**Figure 19:**
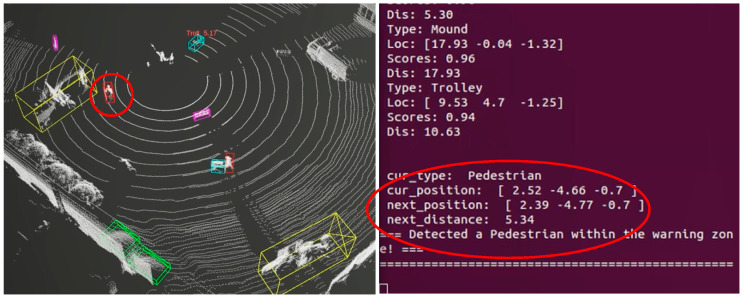
Pedestrian enters within warning threshold.

In order to verify the rationality and feasibility of the early warning braking strategy, the test is carried out. As shown in Figure 19, when the sensing system is running, a staff member is about to approach the excavator; according to the trajectory tracking, the system predicts that the target position of the next frame is in the range of the warning safety distance. At this time, the sensing system immediately sends out an early warning alert, suggesting that there are pedestrians in the non-safety zone, and sends the current position of the target and the predicted position of the next frame in real time to the visualization of the interactive interface.

**Figure 20:**
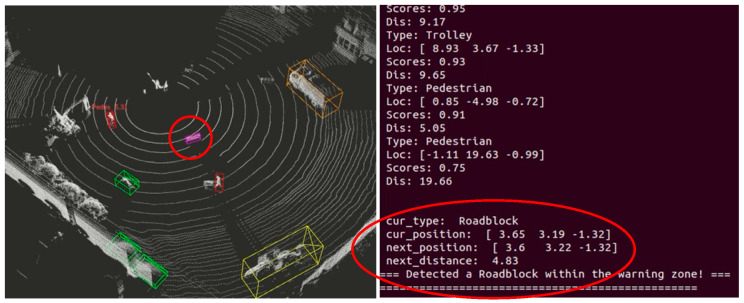
Entering within roadblock warning thresholds.

After the staff leaves the warning threshold range, the sensing system resumes normal operation. When the staff continues to drive into the warning threshold range of the roadblock, as shown in Figure 20, the sensing system sends the current position information of the roadblock as well as the relative predicted position of the next frame to the visualization interactive interface, and sends out a warning alert to inform that there is an obstacle in front of them.

**Figure 21:**
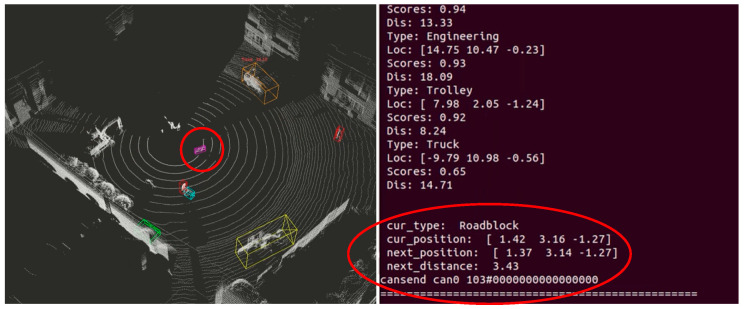
Entering the barricade braking threshold range.

As shown in Figure 21, when the excavator enters the braking threshold range of the barricade, i.e., there is a possibility of collision at the next moment; at this time, the sensing system sends the zero duty cycle message to the proportional decompression valve through the way of CAN bus communication, so that the valve opening is closed, and the left and right track speeds are both set to zero, so as to achieve the purpose of emergency braking, and prevent the next moment collision from happening. The next moment collision occurs, to ensure the safety of the excavator itself and other objects in the vicinity.

## 6. Conclusions

Aiming at the unique challenges posed by unstructured scenes and special targets in construction machinery environments, and to address the scarcity of relevant open-source datasets, this paper constructed a dedicated data collection platform to develop a dataset customized for engineering scenarios. Focusing on the perception layer requirements for environmental sensing in these complex settings, we proposed a LiDAR-based system, which adopts the detection-based tracking paradigm. Based on the predicted trajectories of dynamic targets, a safety warning strategy was developed and demonstrated as a key application of the perception output. In order to verify the effectiveness of the proposed sensing system and dynamic target trajectory prediction, a test platform was built and experimental verifications were carried out. The experimental results demonstrated that the proposed system is better adapted to the specific needs of construction machinery applications, providing effective environmental perception capabilities in complex, unstructured environments. This enhanced perception supports the development of safer and more autonomous operations for construction machinery, by improving situational awareness for operators and enabling functionalities like dynamic obstacle avoidance verification. Most importantly, this work, implemented within the ROS framework, explicitly focuses on attempts to apply perceptual algorithms rather than normative system architecture that fulfill standard safety requirements, and hopefully provide direction for subsequent research related to the automation of construction machinery.

## Figures and Tables

**Table 1:** 3D Target Detection Network Detection Accuracy for different categories.

Categories	Threshold	AP
Engineering	0.7	76.35
Truck	0.7	84.17
Pedestrian	0.35	71.46
Trolley	0.5	71.03
Mound	0.5	78.18
Roadblock	0.5	80.38
mAP	-	76.93

**Table 2:** Comparison with other algorithms.

Meth	mAP	Bloc	Ped	Tro	Mou	Eng	Tru
PP	73.2	***82.7***	47.7	68.0	78.5	76.3	***85.8***
SEC	67.0	64.9	54.0	57.3	74.4	71.4	80.0
CPV	61.8	54.2	27.8	59.2	77.9	72.2	79.7
CPP	68.4	67.5	31.7	72.8	***80.8***	73.7	84.0
VoN	75.4	78.1	67.9	67.3	78.2	***77.7***	83.1
**Our**	***76.9***	80.4	***71.5***	***71.0***	78.2	76.4	84.2

**Table 3:** 3D Target Detection Network Detection Accuracy.

H_RANSAC Statistics Filter	IoU Loss	Weight Coefficient	mAP
			60.63
√			75.38
√	√		76.22
√	√	√	**76.93**

**Table 4:** Trajectory management thresholds.

Categories	Tracking Threshold	Deleting Thresholds
Engineering	3	4
Truck	3	4
Pedestrian	3	4
Trolley	1	2
Mound	1	2
Roadblock	1	2

**Table 5:** Maximum length and width statistics and thresholds for markup boxes.

Category	Maximum L (m)	Maximum W (m)	$\mathrm{Warning}\;S_{w}$	$\mathrm{Braking}\;S_{b}$
Eng	6.61	2.85	9	6
Tru	5.41	2.37	9	6
Ped	1.01	0.86	9	4
Trol	1.83	0.85	6	4
Mou	2.05	3.26	6	-
Roadb	0.41	1.56	6	4

**Table 6:** Specific configuration of the test environment.

Environment	Trial	Deployment
Processors	RTX 3090	Jetson AGV Orin
Ubuntu	18.04	20.04
ROS	Melodic	Noetic
CUDA	11.1	11.4
Python	3.8	3.8
PyTorch	1.8.1	1.11.2

**Table 7:** Excavator Motion Mode.

**Mode**	**Velocity**
Slow (turtle gear)	2.5 km/h
Fast (rabbit gear)	3.8 km/h

**Table 8:** Comparative statistics of target detection at different speeds modes.

Mode	Category	GT/pc	Det/pc	Rate (%)	Average (%)
Fast	Eng	20	19	95.00	82.03
	Tru	20	15	75.00	
	Trol	21	15	71.43	
	Roadb	57	45	78.95	
	Ped	42	30	71.43	
	Mou	57	54	94.74	
Slow	Eng	40	33	82.50	84.05
	Tru	8	6	75.00	
	Trol	34	29	85.29	
	Roadb	55	43	78.18	
	Ped	60	49	81.67	
	Mou	60	56	93.33	

**Table 9:** Comparative statistics of target detection under different motion states.

Mode	Category	GT/pc	Det/pc	Rate (%)	Average (%)
Straight	Eng	40	31	77.50	87.80
	Tru	17	17	100.00	
	Trol	31	29	93.55	
	Roadb	53	42	79.25	
	Ped	53	46	86.80	
	Mou	60	58	96.67	
Turns	Eng	28	23	82.14	88.42
	Tru	20	16	80.00	
	Trol	32	29	90.63	
	Roadb	28	28	100.00	
	Ped	57	53	92.98	
	Mou	25	19	76.00	
Rotation	Eng	30	23	76.67	81.78
	Tru	20	16	80.00	
	Trol	40	30	75.00	
	Roadb	48	42	87.50	
	Ped	60	48	80.00	
	Mou	27	25	92.59	

## Data Availability

The data is available upon reasonable request.
